# Dynamics of clinical and psychological indicators of patients with anxiety neurotic disorders in the treatment of short-term individual analytic-cathartic therapy

**DOI:** 10.1192/j.eurpsy.2021.2078

**Published:** 2021-08-13

**Authors:** E. Ilchenko, T. Karavaeva, E. Abritalin

**Affiliations:** 1 Department Of Psychotherapy, Medical Psychology And Sexology, FGBOU VO “North-West State Medical University named after I.I. Mechnikov”, St. Petersburg, Russian Federation; 2 Day Hospital №1, SPB GBUZ “Psychiatric hospital №1 named after P.P. Kashchenko”, St. Petersburg, Russian Federation; 3 Department Of Treatment Of Borderline Mental Disorders And Psychotherapy, FGBU “National Medical Research Center for Psychiatry and Neurology named after V.M. Bekhterev “of the Ministry of Health of Russia, St. Petersburg, Bekhtereva street, Russian Federation

**Keywords:** analytical-cathartic therapy, ACTA, psychotherapy, neurotic disorders

## Abstract

**Introduction:**

Analytical-cathartic therapy (ACTA), a modern model of psychotherapy, the theoretical basis of which is the psychology of relations of V.N. Myasishchev. ACTA is intended for the treatment of emotional disorders; the study of the dynamics of clinical and pathopsychological characteristics has not been previously conducted.

**Objectives:**

To assess the dynamics of the clinical and pathological characteristics of patients with neurotic disorders, in whose clinical picture anxiety syndrome predominated, in the process of an individual ACTA.

**Methods:**

A specially designed semi-structured interview, HARS, and the CGI (CGI-S - disease severity, CGI-I - improvement dynamics). The study group (N = 90) included patients with neurotic disorders, whose clinical picture was dominated by anxiety syndrome

**Figure:**
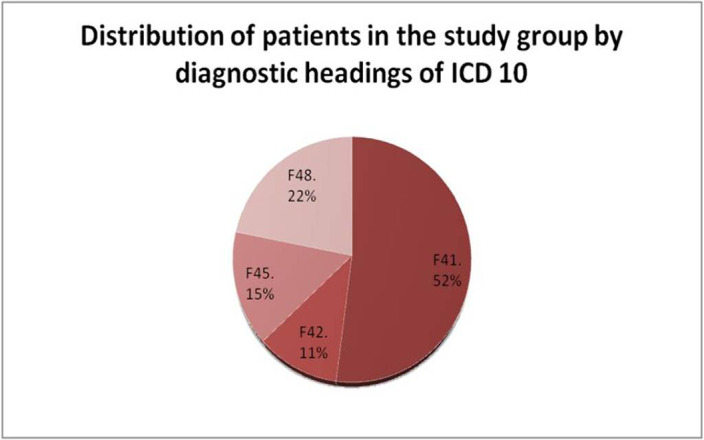

AKTA course 12 sessions 3 times a week for 60 minutes. Psychopharmacotherapy during the period of psychotherapeutic treatment in patients was not carried out.

**Results:**

The study of ACTA was carried out before, after treatment and in the follow-up.

**Figure:**
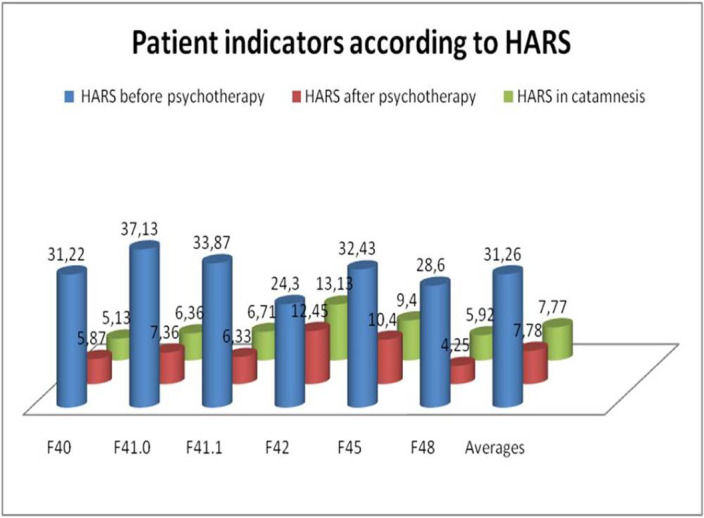

The overall decrease in the HARS score was 73.8% - the high success of the therapy in relation to the reduction of anxiety.

**Figure:**
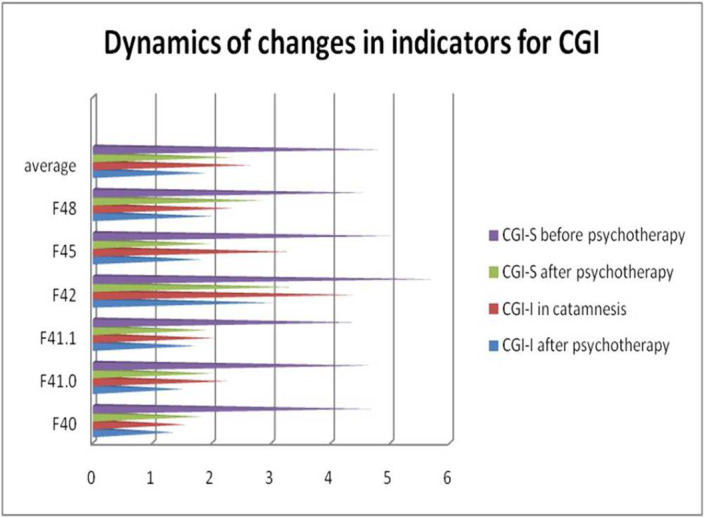

On the CGI-I scale - improvement from minimal to significant. On the CGI-S scale, no more than mild disease severity (p <0.01). The change in the CGI scale also indicates the success of the therapy.

**Conclusions:**

As a result of the study, clinical indicators were determined, on the basis of changes in which in dynamics the success of the treatment can be assessed. ACTA allows to clearly reduce anxiety and improve the general condition of patients with neurotic disorders.

**Disclosure:**

No significant relationships.

